# Unraveling the Enigmatic Behavior of *Cutibacterium acnes*: Exploring Clinical Correlations and Behaviors of Clinical Strains in Prosthetic Joint Infections

**DOI:** 10.1155/ijm/8475639

**Published:** 2026-03-05

**Authors:** Mongaret Céline, Varin-Simon Jennifer, Ohl Xavier, Fulbert Baptiste, Gangloff Sophie, Kanagaratnam Lukshe, Reffuveille Fany

**Affiliations:** ^1^ Pharmacy Department, Clermont-Ferrand University Hospital (CHU), Clermont-Ferrand, France; ^2^ University of Reims Champagne-Ardenne–UR BIOS, Biomaterials and Inflammation at Bone Sites, Reims, France; ^3^ Orthopedic and Traumatology Surgery Department, Reims University Hospital (CHU Reims), Reims, France; ^4^ Pharmacy Department, Reims University Hospital (CHU Reims), Reims, France; ^5^ Department of Research and Public Health, Reims University Hospital, Aging, Frailty (VieFra), Faculty of Medicine, University of Reims Champagne-Ardenne, Reims, France, univ-reims.fr

**Keywords:** *Cutibacterium acnes*, prosthesis joint infection, behavior

## Abstract

**Abstract:**

*Cutibacterium acnes* is an anaerobic bacterium isolated from prosthetic joint infections (PJI), an infection which does not induce clinically relevant symptoms for patients without fever, serum inflammatory markers and has a very indolent course. *C. acnes* species participates in the balance of skin microbiota but is also responsible for infections; this species is regarded as an opportunistic pathogen or pathobiont. The aim of this study was to determine the existence of a correlation between clinical infectious characteristics of patients and *C. acnes* clinical strains behaviors. They were evaluated through the determination of bacterial internalization, persistence rate into osteoblast‐like cells, and biofilm formation capacity before interaction and for internalized bacteria. This phenomenon could play a role in infections without having yet been observed *in vivo*. A total of 28 clinical strains were isolated and analyzed from patients with *C. acnes* PJI. Similar infectious clinical characteristics were observed among the PJI patients, whereas the associated clinical strains have various and heterogeneous behaviors in the *in vitro* assay of this study. Most of the tested *C. acnes* strains (75%) were internalized into osteoblast‐like cells with a higher rate of *C. acnes* strains with phylotype IA1 than other phylotypes (IB and II). High internalization rates of *C. acnes* in osteoblast‐like cells seemed to be associated with strains isolated from patients with no local inflammatory symptoms, especially articular stiffness profile. All the strains were able to form biofilm, and internalization into osteoblast‐like cells modified the capacity of clinical strains to form biofilm significantly for seven clinical strains (25%), associated with the presence of a high level of polymorphonuclear leukocytes‐patient blood with PJI from whom these strains were isolated. In our cohort, the persistence rate of *C. acnes* strains in osteoblast cells is less important for strains isolated from patients with tobacco use. This study raises the hypothesis that the interaction between bone environment, host, and strain modulates *C. acnes* ability to stimulate inflammatory symptoms in patients with *C. acnes* PJI.

**Trial Registration:**

ClinicalTrials.gov identifier: NCT03950063

## 1. Introduction

*Cutibacterium acnes* is a gram‐positive anaerobic aerotolerant bacillus, involved in the human skin health balance by having a defensive role against the environmental pathogens [1, 2]. Interaction of *C. acnes* with the human host, promote the selection of *C. acnes* strains capable of producing several virulence factors that increase inflammatory capability [2]. Its pathogenic role has been conclusively established in multiple forms of chronic infections, with a particular prevalence in prosthetic joint infections (PJI). *C. acnes* is the most frequent anaerobic bacterium and the fourth most frequent species isolated from chronic PJI [3–5], representing up to 50% of monomicrobial prosthetic shoulder infections [6, 7]. Most *C. acnes* associated orthopedic implant infections (82%) appear late after implantation, mostly classified as delayed (3–24 months) or late infections (> 24 months) [8, 9]. Specific bacteriological culture conditions of the *C. acnes* species like extended incubation under anaerobic environment increases the difficulties to identify this infection [9–11] but the main reason is that these species lead to low‐grade clinical symptoms. Fever is typically absent, and local inflammatory signs or the presence of a fistula are observed in fewer than one‐third of patients with hip or knee infections and serum inflammatory markers such as C‐reactive protein (CRP) or polymorphonuclear leukocytes can be normal or only slightly elevated [9–11]. The most constant symptom for patients with *C. acnes* PJI is persistent joint pain and a prosthesis dysfunction.

PJI are associated with substantial patient comorbidities, some of whom increase the risk of PJI, especially diabetes mellitus, obesity, and smoking [12]. The increasing number of joint replacements has engendered an increased PJI incidence, with significant impact on the devastating complication of joint‐replacement surgery and patients′ morbidity [13]. Thus, PJI have an important economic burden worldwide, as described by Grammatico‐Guillon et al. in France [14, 15].

*C. acnes* infections do not elicit a pronounced inflammatory response, indicating that this microorganism can attenuate and/or evade the immune system, likely by disrupting host–pathogen interactions through its ability to internalize within host cells, for example [16]. Some studies have already demonstrated that *C. acnes* can be internalized into bone‐resorbing cell lines such as osteoclasts derived from CD14+ monocytes and bone‐forming cell lines such as MG63 or SaOS‐2 cell lines [17–19]. Moreover, bone marrow‐derived MSCs appear to provide a niche for *C. acnes*, but their intracellular rate is higher than MG63′s, whose internalizing *C. acnes* rate is around 1% [20]. The interaction in bone cells modified the behavior of noninfectious *C. acnes* strains, of which 50% of the strains demonstrated a significant enhancement of biofilm formation following internalization by osteoblast‐like cells (2.8‐fold increase). The biofilm formation is often identified as the origin of persistent and difficult‐to‐treat infection [21]. This biofilm‐forming ability was substantially higher on titanium than on plastic surfaces, irrespective of interaction with SaOS‐2 cells. Specifically, a 22‐fold and 21.6‐fold increase in biofilm formation on titanium compared with plastic were observed before and after internalization, respectively [18]. Moreover, Dubus et al. showed that internalization of *C. acnes* with bone marrow mesenchymal stem cells induced modifications in *C. acnes* wall characteristics [20]. The internalization of *C. acnes* in two different cell types influences biofilm formation depending on the strain and material [18, 20].

*C. acnes* participating in the balance of skin microbiota, but also responsible of infection, can be qualified as opportunistic pathogen or pathobiont, as described as [22]. However, the complexity of microbiota/host interactions is so complex that this term might be too simple [23]. *C. acnes* virulence factors are not well‐described except those identified as involved in biofilm formation (*e.g.,* FnBP or DsA1 adhesins) [24, 25] or interaction with host cell (*e.g.,* CAMPs) [26]. Different *C. acnes* phylotypes are described with a predominance of phylotype IA1 and IB lines in PJI whereas Phylotype III line has never been identified in PJI [5, 27]. Thus, the diagnosis is difficult and predicting the outcome of an infection based on the *C. acnes* strain type currently appears complicated [28].

The aim of this study was to determine the existence of a correlation between clinical infectious characteristics of patients with *C. acnes* PJI and *C. acnes* clinical strains behaviors to predict infection outcome and adapt therapeutic strategy. They were evaluated through the determination of their internalization and persistence rate into osteoblast‐like cells, as well as the biofilm formation capacity before interaction and for internalized.

## 2. Material and Methods

### 2.1. Ethic Statement

The Reims University Hospital (CHU de REIMS) has an authorization from the National Commission for Computing and Freedoms (CNIL) through the reference methodology MR004 to comply with the provisions of the Data Protection Act and the European provisions of the General Regulations on Data Protection (CNIL MR004 Conformity Number: 2206749 v 0). All patients received a written nonopposition document. All data were anonymous.

### 2.2. Study Design/Inclusion/Exclusion Criteria

This is an observational retrospective monocentric cross‐sectional study. Briefly, we retrospectively included patients with a *C. acnes* PJI between December 2016 to July 2020.

The eligibility criteria of patients were described in Clinicaltrials.gov as follows: with a diagnosis of *C. acnes* prosthesis and joint infection whatever the site (hip, knee, shoulder, and spine) (at least three positive of the five samples from the bone and joint tissue sampled during orthopedic surgery); with ≥ 18 years old; viable bacterial strain during defrosting; and the presence of a single species.

### 2.3. Data Collection of Patients With a *C. acnes* PJI

Data from patients were retrospectively collected using a standardized data collection sheet. Clinical features for patients included were collected including patient characteristics (sex, age, body mass index, active tobacco, and diabetes mellitus), site of PJI, time to occurrence from prosthesis implantation, clinical inflammatory markers such as fever, local inflammation symptoms (redness, joint pain, and stiffness), bone infection signs (fistula, purulent wound, and luxation), and serum inflammatory markers such as leukocyte count, PMN count, and CRP level.

### 2.4. Pathogenicity Factors of *C. acnes* Clinical Strains

#### 2.4.1. Bacterial Strains and Culture

Clinical *C. acnes* strains resulting from a monomicrobial infection, which were isolated and collected at the laboratory of bacteriology of Reims University Hospital Center (CHU Reims) were retained for this study. Then 28 clinical *C. acnes* strains, which had resumed growth after storage were included for this study. In this study, *C. acnes* was isolated on Columbia agar with 5% sheep blood (BioRad, Hercules, California, United States) under anaerobic conditions using the GenBox system (bioMérieux, Marcy l′Etoile, France) at 37°C for 5 days. A 5‐day culture allowed all strains in the study to grow sufficiently to reach an absorbance > 1. After anonymization, a molecular typing method was performed to determine the ST of each strain following the MLST8 model. Briefly, the DNA of *C. acnes* was extracted and genes (*aroE*, *atpD*, *gmk*, *guaA*, *lepA*, *soda*, *tly*, and *CAMP2*) were amplified by PCR. The samples as well as PCR primers were then sent to Genewiz (Leipzig, Germany) for sequencing. Results obtained were analyzed using the online site PubMLST (https://pubmlst.org/bigsdb?db=pubmlst_pacnes_seqdef).

#### 2.4.2. Cell Culture Media

The human osteosarcoma cell line SaOS‐2 (ATCC HTB‐85) was obtained from ATCC and cultured in Dulbecco′s Modified Eagle Medium (DMEM, Gibco, Invitrogen, Carlsbad, California, United States) supplemented with 10% fetal bovine serum (FBS, PAN‐Biotech GmbH, Aidenbach, Germany) and 1% antibiotic PenStrep solution (Gibco, Invitrogen, Carlsbad, California, United States) at 37°C, 5% CO_2_ humidified atmosphere. The medium was changed every 2 days.

#### 2.4.3. *C. acnes* Internalization by Osteoblast Cells

Bacterial internalization experiments were adapted from the protocol described by Josse et al. [18]. Briefly, SaOS‐2 cells were seeded at a density of 10.5 × 10^3^ cells/cm2 in 24‐well culture plates and incubated at 37°C for 72 h. After incubation, cell cultures were washed with phosphate‐buffered saline (PBS; Gibco, California, United States) and subsequently incubated overnight with 1 mL of antibiotic‐free medium. The following day, cells were washed again with PBS and replenished with 1 mL of antibiotic‐free medium. One well was used to determine the number of cells per well. Bacterial suspensions were centrifuged for 5 min at 5000× g and the pellets were rinsed twice with PBS. Absorbance was then measured, and bacteria were diluted in 1× PBS before being added to the culture medium to achieve a theoretical multiplicity of infection (MOI) of 100:1 (*C. acnes*:cell). After 3 h of interaction, cells were washed twice with PBS and incubated with cell medium containing 100 *μ*g/mL of gentamicin (Fisher Scientific, Hampton, New Hampshire, United States), during 1 h at 37°C, in a 5% CO_2_ humidified atmosphere. Gentamicin, used at a concentration higher than the MIC determined in the literature [29], was selected because this hydrophilic molecule cannot cross the plasma membrane and therefore remains in the extracellular compartment [30, 31]. Cells were then washed twice with 1× PBS and either maintained in culture for 48 h or lysed with 0.1% Triton X‐100 (Sigma, Saint Louis, Missouri, United States) to harvest intracellular bacteria. Lysates were plated on blood agar using the automatic seeder easySpiral (Intersciences, St Nom de la Breteche, France) in exponential mode, covering up to 4 log CFU (colony‐forming units). Plates were incubated at 37°C under anaerobic conditions using the GenBag system for 5 days to determine the number of recovered CFU. The percentage of bacteria was calculated as follows:

Percentage of bacteria=CFU/mL×100number of cells×theoretical MOI.

#### 2.4.4. Persistence of Intracellular *C. acnes* Into Osteoblast‐Like Cells

After 48 h of culture in cell medium without antibiotics (time before change of the medium of osteoblast‐like cells), the supernatant of infected cells was seeded on blood agar plates to quantify bacteria released in medium and intracellular bacteria were harvested by lysing cells using Triton. The percentage of the persistence of intracellular *C. acnes* or the releasing in the supernatant was determined by normalizing to the percentage of *C. acnes* internalization after 3 h. Each experiment was performed independently three times, with at least two technical replicates per experiment, resulting in a minimum of six raw data points for statistical analysis.

#### 2.4.5. Static Biofilm Model (Cristal Violet Staining Model)

The biofilm biomass was assessed using crystal violet staining [19, 20]. An isolated colony of *C. acnes* was inoculated into 1 mL of BHI medium in a 48‐well plastic microtiter plate. After 5 days of anaerobic incubation at 37°C, all strains grew sufficiently to reach an absorbance value > 1. Under these conditions, the planktonic phase was typically achieved by Day 3, whereas biofilm formation was complete by Day 5. The medium was eliminated and the plates were gently washed three times with water to eliminate planktonic aggregates before staining with 1 mL of 0.18% crystal violet (bioMérieux, Marcy l′Etoile, France), in the dark, for at least 20 min. After three washes with water, 1 mL of 95% ethanol was added to each well and the stained biofilm was evaluated by measuring the absorbance at 595 nm. All results are presented after subtracting the blank control, consisting of medium without bacteria. The biofilm biomass was quantified for each *C. acnes* strain before interaction and after being internalized by osteoblast‐like cells (biofilm formation of internalized bacteria) to evaluate the impact of internalization on *C. acnes* behavior. Each experiment was done three (independent) times and with at least three technical repeats (at least nine raw data points).

#### 2.4.6. Graphical Representation and Statistical Analysis

##### 2.4.6.1. Statistical Analysis.

Descriptive analysis was performed; qualitative variables were described as number and percentage and quantitative as median and interquartile range (1st quartile = Q1; 3rd quartile = Q3) or minimum and maximum or mean (standard deviation).

For *in vitro* experiments, all values represent the means of at least three independent experiments (biological replicates), with each experiment including a minimum of three technical replicates (yielding six to nine raw data points). Statistical significance was assessed using nonparametric analyses with pairwise comparisons. Specifically, the Wilcoxon–Mann–Whitney test for independent samples was applied (GraphPad Prism v8). Nonparametric methods were selected due to the nonnormal distribution of the variables. Stratification accounted for technical variability. Differences were considered statistically significant at *p* < 0.05. Data were visualized using GraphPad Prism v8. Bivariate analysis was performed between clinical data (sociodemographic, clinical inflammatory data) and specific pathogenicity factors of *C. acnes* clinical strains using the Mann–Whitney test.

Analyses were performed using SAS Version 9.4 (SAS Institute).

## 3. Results

### 3.1. Clinical Characteristics of Patients Enrolled

A total of 28 patients with a mean age of 57 years (min = 18–max = 89) were analyzed. Patients were predominantly male with a sex ratio (F/H) of 0.4. Most patients had overweight (*n* = 10) and obesity (*n* = 10) and the BMI median of patients was 28.68 kg/m^2^ (IQR = 23.43–30.64). Ten patients had an obesity including two with a severe obesity (BMI > 35 kg/m^2^). Four patients were active smokers. Site of *C. acnes* PJI are on the shoulder for 43% of patients (*n* = 12). *C. acnes* infection appeared 319.5 days after the prosthesis was implanted [IQR : 37.5–763.5]. *C. acnes* infection appeared more than 3 months later the surgery for 23 patients (79%). No patient had fever. Most patients had at least one local inflammatory symptom such as local pain (20 patients) but only five patients had an erythema as local inflammation symptom. All patients had a mean of blood white cell count of 8.5 G/L (IQR = 7.1–10.3) and PMN 5.3 G/L (IQR = 4.3–7.6). Blood white cell count and PMN were increased for only two patients with, respectively, 11.6 and 15 G/L (> 11.5 G/L) and 9.4 and 11 G/L (> 7.5G/L). Ten patients had a value of CRP > 5 mg/L. Clinical characteristics of patients were described in Table1.

**Table 1:** Demographic, clinical, and biological characteristics of patients with *C. acnes* prosthesis joint infection (PJI). WBC, white blood cells; PMN, polymorphonuclear cells; CRP, C reactive protein.

	*n* = 28 (%)

Male^a^	20 (71.4)
Age ≥ 65 ans^a^	14 (45.0)
BMI abnormal (kg/m^2^) (< 18,5 et > 25)^a^	20 (71.4)
Active smoking^a^	4 (14.8)
Diabetes mellitus^a^	6 (22.2)
Primary arthroplasty^a^	7 (24)
≥ 1 revision on implant^a^	6 (21)
Site of surgery^a^	
Hip	4 (14.3)
Shoulder	12 (42.9)
Knee	4 (14.3)
Spine	8 (28.6)
Time occurrence with prosthesis implantation > 30 days^a^	23 (79)

**Clinical inflammatory characteristics**	

Fever^a^	0
At least one of local inflammation signs (erythema, joint pain, and stiffness)^a^	21 (77.8)
At least one of bone infection signs (fistula, purulent wound, and luxation)^a^	14 (51.8)

**Laboratory parameters**	

WBC count, G/L^b^	8.5 (7.1–10.3)
PMN, G/L^b^	5.3 (4.3–7.6)
CRP, mg/L^b^	17 (5.5–54)

^a^Data are the number (%) of patients.

bMedian (1st and 3rd IQR, interquartile ranges).

### 3.2. *In Vitro* Study of *C. acnes* PJI Clinical Strains Behavior

We investigated the phylogenetic profile of *C. acnes* isolated from 28 PJI clinical strains. We revealed in Table2 that 64% (*n* = 18) isolated form PJI belonged to phylotype IA1, the predominant phylotype whatever the origin of the infection (shoulder, spine, knee, and hip), eight to Type IB and only two to Phylotype II (knee and shoulder clinical origin).

**Table 2:** Multilocus sequence typing (MLST) profiles and phylotypes of 28 *C. acnes* clinical strains.

Isolate name	Clinical source	Sequence typing	Clonal complex MLST	Phylotype
PJI1	**Shoulder**	20	CC1	IA1
PJI2		1	CC1	IA1
PJI3		20	CC1	IA1
PJI32		19	Singleton	IA1
PJI27		19	Singleton	IA1
PJI39		19	Singleton	IA1
PJI6		51	Singleton	IB
PJI8		152	CC5	IB
PJI11		5	CC5	IB
PJI21		42	CC5	IB
PJI34		5	CC5	IB
PJI40		27	Singleton	II

PJI4	**Spine**	21	CC4	IA1
PJI18		49	CC1	IA1
PJI22		143	CC1	IA1
PJI30		19	Singleton	IA1
PJI31		19	Singleton	IA1
PJI41		143	CC1	IA1
PJI42		20 or 101 or 127^a^	CC1	IA1
PJI44		228 or 241^a^	CC5	IB

PJI9	**Knee**	152	CC5	IB
PJI26		20	CC1	IA1
PJI29		20	CC1	IA1
PJI36		135	CC6	II

PJI7	**Hip**	55	CC4	IA1
PJI28		18 or 43^a^	CC3	IA1
PJI33		5	CC5	IB
PJI43		20	CC1	IA1

^a^Strain for which multiple ST matches have been identified.

Bacteria can develop persistent behavior like the internalization in bone cells and biofilm formation, assessed in this study for all clinical PJI strains in *in vitro* models.

**Figure 1:**
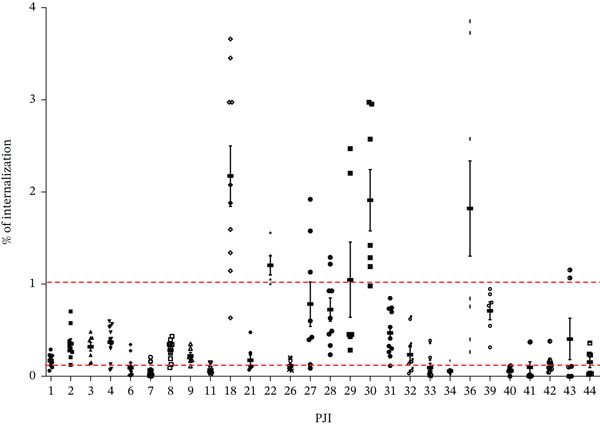
Internalization rate of the PJI *C. acnes* strains by osteoblast‐like cells (SaOS‐2). Red dotted line represented internalization rate of 0.01% and 1%. Each experiment was done three (independent) times and with at least three technical repeats (at least nine raw data points).

Firstly, the internalization rate of 28 clinical PJI strains by osteoblast‐like cells (SaOS‐2) was evaluated. Most of the PJI clinical strains (57%) had an internalization rate between 0.1% and 1% (16/28). Five clinical strains had an internalization rate more/higher than 1% (PJI18, PJI22, PJI28, PJI30, and PJI36) as shown in Figure 1. Conversely, seven clinical strains did not internalize into osteoblast‐like cells (internalization rate < 0.1%).

Then, the capacity of biofilm formation of all the strains was determined by crystal violet staining.

**Figure 2:**
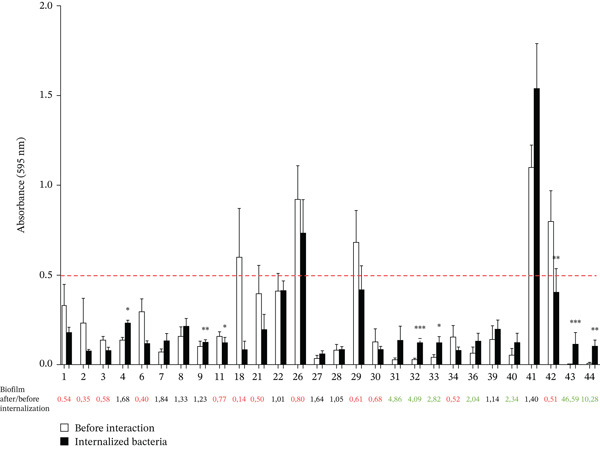
Biofilm formation of PJI *C. acnes* staining by crystal violet. Biofilm forming capacity by crystal violet quantification before interaction (white bars) and after internalization for internalized bacteria (black bars) in osteoblast‐like cells.  ^∗^p < 0.05;  ^∗∗^p < 0.01; ∗∗∗*p* < 0.001. Red dotted line represented biofilm quantification by crystal violet at 0.5 of absorbance (595 nm). All results are expressed with the subtraction of the blank: medium without bacteria. Each experiment was done three (independent) times and with at least three technical repeats (at least nine raw data points).

*C. acnes* strains isolated from periprosthetic joint infections (shoulder prostheses or osteosynthesis material) were evaluated for their biofilm‐forming capacity both prior to interaction with osteoblast‐like cells and after internalization (biofilm formation of internalized bacteria). In the basal state, five clinical strains (18%) formed a high amount of biofilm with an average of absorbance > 0.5 (PJI18, PJI26, PJI29, PJI41, and PJI42) (Figure 2, white bars).

To assess whether internalization in osteoblast‐like cells affects *C. acnes* biofilm‐forming capacity, internalized bacteria were collected and cultured, and biofilm formation was monitored after 5 days of incubation. After internalization (Figure 2, black bars), a change of *C. acnes* behavior was observed by calculating the ratio of biofilm formation of internalized bacteria/before interaction bacteria. We observed for 12 *C. acnes* clinical strains (43%), a decrease of the biofilm formation for internalized bacteria with a significant decreased for two clinical strains (PJI11; PJI42). Inversely, 16 clinical strains (57%) formed more biofilm for internalized bacteria into osteoblast like cells with a significant difference for five clinical strains. Thus, internalization into osteoblast‐like cells modified the capacity of clinical strains to form biofilm significantly for seven clinical strains (25%).

**Figure 3:**
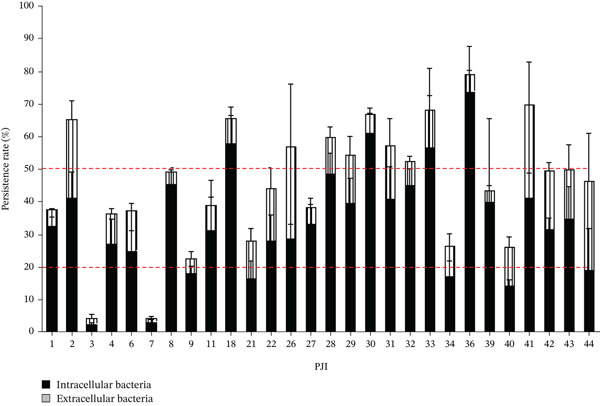
Persistence rate of PJI *C. acnes* after internalization in osteoblast‐like cells. The percentage of the persistence of intracellular *C. acnes* or the releasing in the supernatant was determined by normalizing to the percentage of *C. acnes* internalization after 3 h. The red dotted line represented the persistence rate of 20% and 50%. Percentage of bacteria in supernatant after 48 h (extracellular bacteria) (black bars). Percentage of intracellular bacteria after 48 h (stripe bars). Each experiment was done three (independent) times and with at least three technical repeats (at least nine raw data points).

Finally, the potential persistence of *C. acnes* in osteoblast‐like cells was estimated by quantifying the number of intracellular *C. acnes* 48 h postinternalization but also the quantity of released *C. acnes* (Figure 3). Overall, most strains (23/28) persisted in cells with a percentage of intracellular bacteria between 20% and 50%. However, a part of the bacteria was externalized with a mean of 11.1% (1.9% [PJI3]–28.5% [PJI41]). Another part is supposedly dead as it was not found in the cytoplasm or in the extracellular medium. Surprisingly, two strains did not seem to have the same capacity to persist with a percentage of intracellular bacteria less than 10 (PJI3 and PJI7).

### 3.3. Clinical Characteristics of Patients Associated With *C. acnes* PJI Behavior

The potential correlation between clinical characteristics of patients with *C. acnes* PJI and these clinical strains microbiological behavior was evaluated in Table 3 *. C. acnes* clinical strains with Phylotype IA1 (64%), which is the most frequent in PJI as described, had significantly more internalization rate into osteoblast like cells than clinical strains with Phylotypes IB (29%) and II (7%) with a mean of 0.39 and 0.125, respectively (*p* = 0.022).

**Table 3:** Clinical characteristics of patients with a *C. acnes* prosthesis joint infection (PJI) associated with their *C. acnes* strains′ behavior.

	**Internalization rate *median (intervalle quartile)***	**p**

Female	0.39 (0.23–1.48)	0.138
Male	0.2 (0.10–0.59)	
Local inflammation symptoms	0.22 (0.10–0.41)	0.127
No local inflammation symptoms	0.64 (0.18–1.91)	
Local pain	0.19 (0.10–0.37)	0.147
No pain	0.41 (0.18–1.21)	
Articular stiffness	0.14 (0.08–0.34)	0.099
No articular stiffness	0.37 (0.15–1.05)	
Shoulder	0.21 (0.08–0.33)	0.143
Other sites	0.39 (0.13–1.13)	
Phylotypes Ib et II	0.125 (0.07–0.22)	0.022
Phylotype IA1	0.39 (0.17–0.78)	

	**Ratio biofilm quantification post/pré internalization**	**p**

PMN abnormal	2.06 (1.01–2.8)	0.08
PMN normal	0.89 (0.52–1.6)	

	**Intracellular bacteria rate after 48 h**	**p**

Active smoking	14.3 (3.05–33.2)	0.07
No smoking	35.0 (28.2–48.8)	
Diabetes mellitus	43.4 (32.6–50.0)	0.149
No diabetes mellitus	31,8 (18.4–41.4)	

Clinical strains that did not induce local inflammatory symptoms on patients infected, especially joint stiffness, tended to internalize higher into osteoblast‐like cells than clinical strains that cause at least one local symptom (0.34–0.17, *p* = 0.09) suggesting a potential correlation between internalization rate into osteoblast‐like cells and local clinical symptom of patients with a *C. acnes* PJI. Clinical strains from patients with systemic abnormal PMN (7%) showed an increase in biofilm formation for internalized bacteria with a median ratio post/preinternalization of 2.6 for patients with abnormal PMN compared with patients with normal PMN with a median ratio of 0.89 (*p* = 0.08) suggesting that clinical strains that formed more biofilm after internalization into osteoblast‐like cells are correlated with patients with abnormal PMN. Surprisingly, *C. acnes* clinical strains of patients with active tobacco had a persistence rate in osteoblast‐like cells less important (14.3%) than nonsmoker patients (35.0%) (*p* = 0.07) suggesting a potential impact of nicotine on persistence rate of *C. acnes* strains into osteoblast‐like cells.

## 4. Discussion

To our knowledge, this is the first study to evaluate both clinical characteristics of patients with *C. acnes* PJI and microbiological behavior of *C. acnes* clinical strains (internalization rate, capacity to form biofilm, and persistence rate). Similar infectious clinical characteristics were observed among the PJI patients, whereas the associated clinical strains have various and heterogeneous behaviors in the *in vitro* assay of this study. The virulence phenotypes like cell internalization, persistence, or biofilm formation are variables according to clinical strains but would not reflect through clinical symptoms among patients, suggesting an influence of environment on *C. acnes* behavior.

In this case series including 28 patients, there is a predominance of male (20 patients) in our patient cohort with PJI similarly to other reports [9, 13, 32, 33], with a median age of 57 years old [13, 32]. *C. acnes* PJI were characterized by absence of fever. Fever is the first clinical symptom of infection that patients monitor after surgery. This is in accordance with some studies describing clinical symptoms on *C. acnes* chronic infections such as bone and joint infections, shoulder infections, and *C. acnes* endocarditis [34]. A lack of characteristics systemic infectious symptoms leads to difficult and delayed diagnosis. The hypothesis is that immune response is not adapted to *C. acnes* persistence. Moreover, serum inflammatory markers, such as CRP, and white blood cell count, were not highly present in our patient cohort as described in other patients′ cohort with *C. acnes* infection. Serum markers have a low sensitivity to diagnose *C. acnes* infections [35]. However, we noted that a local pain was present in most patients (80%), whereas local signs of inflammation were reported only in 28%.

Most of the tested *C. acnes* strains (75%) were internalized into SaOS‐2 osteoblast‐like cells with a rate ranged between 0.1% and 2.2% but 25% had a rate under 0.1%. Not all strains can be internalized as previously observed into another osteoblast‐like cell line, MG 63 cells, where an internalization rate of 0.1% was determined for four on six clinical PJI strains, whereas two on six strains had an internalization rate < 0.01% [17]. The internalization method provides an estimate of the internalization phenomenon but may under‐ or overestimate the number of internalized bacteria (due to Triton treatment, viable but nonculturable or persistent bacteria, or bacteria adhered to the cell surface and resistant to gentamicin). In this study, the internalization rate is higher and more variable than in Aubin et al. study, probably due to the greater number of tested strains, and due to the different type of cells [17]. This difference may be attributed to different cell culture conditions, using 5% FBS compared with 10% in our study. Moreover, the different rate of internalization through *C. acnes* strains raises numerous hypotheses like, for example, the presence of different structure in the cell wall, leading to a different capacity of bacterial adhesion on bone cells and internalization. The internalization of *C. acnes* has already been reported in various cell types. For example, Dubus et al. [20] showed that after 3 h of interaction, approximately 4% of the bacteria were found within the intracellular compartment of primary human bone marrow‐derived MSCs. It would be interesting to extend these observations by using primary human osteoblasts, which better mimic the bone environment, while considering their culture limitation to three passages.

Our results revealed that 21/28 PJI clinical strains intracellularly persist in osteoblast‐like cells for 48 h (> 20% of intracellular *C. acnes*). *C. acnes* is hypothesized to persist intracellularly in a quiescent state, which can later be reactivated to proliferate at the site of latent infection [36–38]. Hudek et al. suggested that a high persistence rate of *C. acnes* in macrophage and stroma cells in osteoarthritic joints might link its intra‐articular presence to the initiation of osteoarthritis [36]. Dormant forms could be endogenously activated under certain environmental conditions and then proliferate in cells at the site of the latent infection. Through this spread of infective *C. acnes*, a new latent infection might be caused in distant body sites, including the shoulder [37]. Bone environment might influence persistence of *C. acnes* strains.

In our study, the three most frequent phylotypes of *C. acnes* PJI strains were Phylotype IA 1, IB and II. As described in several studies, the Phylotype III was not identified [5, 27]. Regarding the joint site of infection, we identified in our cohort, only one clinical strain with hip origin whereas in Salar‐Vidal et al. cohort of *C. acnes* PJI, the Phylotype IB is predominant [5]. The most common phylotype among the PJIs in our study was Phylotype IA1 as revealed in Liew‐Littorin et al. on *C. acnes* isolated from 55 patients with PJIs [39]. This phylotype is commonly found in the facial area and upper part of the body [27, 40]. These discreprancies between different studies might be due to the types of joints involved, types of infection (monomicrobial or polymicrobial), or geographical origin [5]. In the present study, *C. acnes* strains with Phylotype IA1 were significantly more internalized into osteoblast‐like cells than other phylotypes (IB and II) of *C. acnes* strains. This result confirmed a previous study with a smaller cohort of patients which revealed that lineage of *C. acnes* with a Phylotype IB/CC36 are less internalized in bone cells than those with a lineage of Phylotype IA1 CC18/CC28 [17]. Interestingly, the Phylotype IA1 is the most common phylotype found in PJI and the strains with higher internalization rate. In perspective, RNA sequencing of clinical strains could identify differences in the expression of virulence or adaptation genes. Even if it is not possible to make a clear link between the two statuses, it would be interesting to study a possible correlation between the ability of bacteria to be internalized and persist with the initiation of infection in bone environment.

All the tested strains were able to form a biofilm on plastic, but 18% formed robust biofilms. There is now convincing evidence that *C. acnes* biofilms are involved in infections related to the use of prosthetic joints, such as orthopedic devices [16]. After internalization, modification of the amount of biofilm was observed for seven clinical strains (25%), underlining an impact on biofilm formation of the passage of bacteria into osteoblast‐like cells, as suggested by Mongaret et al. [18]. We found that there is no correlation between biofilm formation and phylotype. Moreover, the bone environment might influence bacterial behavior [41–44]. An increase of biofilm formation after osteoblast‐like internalization was associated with high PMN serum markers suggesting that *C. acnes* biofilm activated PMN and immune response, but it is not sufficient and this PMN activation requires other environmental factors. Overall, we speculate that even transient intracellular survival may select for the most resilient bacteria, potentially influencing their behavior and enhancing their virulence. It would be interesting to prolong the duration of this experiment and evaluate internalized clinical strains behavior (biofilm quantification, SCV …) to evaluate this hypothesis. A high internalization rate of *C. acnes* in osteoblast‐like cells seems to be associated with patients with no local inflammatory symptoms, especially articular stiffness. These results are in accordance with the capacity of *C. acnes* to attenuate immune system response and to persist in the bone environment. The influence of PJI environment on bacterial behavior is described for other bacteria frequently involved in PJI: *S. aureus*. The internalization of *S. aureus* into osteoblast and the secretion of bone cells also influence *S. aureus* biofilm formation under PJI environment [41, 45]. A larger number of patients in our cohort would allow us to verify our results on the bone environment on *C. acnes*.

In our cohort, the persistence rate of *C. acnes* strains in osteoblast‐like cells is less high for strains isolated from patients with tobacco use. Nicotine‐mediated vasoconstriction has been postulated as the main cause for deficient wound healing and bad circulation results in tissue hypoxia and increased infection susceptibility [46]. Several meta‐analyses across several surgical subspecialties have highlighted the benefits of preoperative smoking cessation, reducing postoperative infections by more than 50% [47]. Roach et al. investigated the potential microbial colonization of subscapularis tagging sutures during shoulder arthroplasty, and active tobacco use was associated with positive culture suggesting a potential impact of nicotine on the persistence rate of *C. acnes* strains into osteoblast‐like cells [48]. We speculated that the presence of nicotine could inhibit *C. acnes* persistence but allow the development of bacteria, leading to an increased development of acute infection.

In our study, no statistically significant correlation (*p* < 0.05) was found between the clinical characteristics of patients with *C. acnes* PJI and the microbiological behavior of these clinical strains. Therefore, we did not conclude any correlations, but these are suggested trends that should be confirmed with a larger cohort of patients.

## 5. Conclusion

Similar infectious clinical characteristics were observed among the PJI patients, whereas the associated clinical strains have various and heterogeneous behaviors in the *in vitro* assay of this study.

This study suggests that the interaction between bone environment, host, and strain modulates *C. acnes* ability to stimulate inflammatory symptoms in patients with *C. acnes* PJI. In perspective, a prospective study on *C. acnes* PJI patients collecting clinical *C*. *acnes* strains from different environment (skin, joint, and prosthesis) should allow to compare strains behavior and their genetic profile.

This study suggests that active tobacco, known to have a role on inflammatory symptoms in patients with PJI, influences *C. acnes* persistence in osteoblast‐like cells. This confirms the influence of the host and environment on *C. acnes* behavior.

## Funding

This study was supported by AOL CHU Reims.

## Conflicts of Interest

The authors declare no conflicts of interest.

## Data Availability

The data that support the findings of this study are available from the corresponding author upon reasonable request.
